# Distinct Cerebrospinal Fluid Findings in a Case of 20-Year-Old Woman Patient With Meningoencephalitis

**DOI:** 10.7759/cureus.97661

**Published:** 2025-11-24

**Authors:** Waleed Bandey, Maryam Zafar, Anam Siddique, Nimrat Ul Ain Banday, Mandar Marathe

**Affiliations:** 1 Emergency Medicine, Leicester Royal Infirmary, Leicester, GBR

**Keywords:** cerebrospinal fluid (csf), c-reactive protein (crp), csf glucose, csf protein, meningo-encephalitis, polymerase chain reaction (pcr), xanthochromia

## Abstract

Meningoencephalitis is a critical condition that necessitates prompt diagnosis and intervention. This case report underscores the importance of interpreting xanthochromia in cerebrospinal fluid (CSF) in relation to not only vascular pathology but also infectious and inflammatory conditions. Additionally, it is essential to recognize that a normal glucose level should not eliminate the consideration of meningoencephalitis, particularly when there is a strong clinical suspicion. Lastly, it is important to note that a moderately elevated protein level in the CSF can still be consistent with meningoencephalitis, even if it does not reach the threshold typically associated with bacterial infections.

## Introduction

Meningoencephalitis is a rare and potentially life-threatening condition defined by the simultaneous occurrence of meningitis and encephalitis [1]. Meningitis can be attributed to two primary routes of infection. The first route, known as hematogenous seeding, involves the initial colonization of bacteria in the nasopharynx. Following this, the bacteria breach the mucosal barrier and enter the bloodstream, subsequently reaching the subarachnoid space and crossing the blood-brain barrier. This process triggers a significant inflammatory response within the central nervous system. The second route of infection is direct contiguous spread. In this case, pathogens can access the cerebrospinal fluid (CSF) through adjacent anatomical structures, commonly associated with conditions such as otitis media or sinusitis. Furthermore, bacteria may infiltrate the CSF via foreign objects, including medical devices, penetrating trauma, or during surgical interventions [2].

Encephalitis may occur as a result of direct viral invasion or as a post-infectious immunological complication, which is related to a hypersensitivity reaction to a virus or another foreign protein. The viruses implicated in encephalitis can be classified as either epidemic, such as poliovirus, or sporadic, including herpes simplex and varicella-zoster virus [3]. Meningoencephalitis is primarily attributed to various infectious agents, encompassing viruses, bacteria, fungi, and the amoeba *Naegleria fowleri*. These infections can be transmitted through air, water, food, or close contact with infected individuals. However, not everyone who contracts these infections will develop meningoencephalitis. In some cases, autoimmune factors may contribute to the condition, although the underlying mechanisms remain poorly understood. Risk factors for the development of meningoencephalitis include a personal or family history of autoimmune diseases, younger age, and female gender [1].

Bacterial causes include *Neisseria meningitidis*, *Listeria monocytogenes*, *Rickettsia prowazekii*, *Mycoplasma pneumoniae*, *Mycobacterium tuberculosis*, *Borrelia *(Lyme disease), and *Leptospira* species [4]. Viral causes include tick-borne encephalitis, West Nile virus, measles virus, Epstein-Barr virus, varicella-zoster virus, enteroviruses, herpes simplex virus types 1 and 2, mumps virus, and HIV [5]. Symptoms are fever, headache, stiff neck or neck pain, fatigue, nausea and vomiting, muscle weakness, speech impairment, photophobia, cognitive dysfunction, personality and behavior changes, hallucinations, seizures, and loss of consciousness [1].

Healthcare providers use several tests to confirm a diagnosis of meningoencephalitis. These tests include: (1) Healthcare providers analyze body fluids like urine, blood, and saliva to detect signs of infection, particularly through elevated white blood cell counts. These tests assist in identifying the specific type of infection. (2) A spinal tap involves inserting a needle into the lower back to collect cerebrospinal fluid (CSF) for laboratory testing. The tests check for bacteria, fungi, abnormal cells, protein types and levels, and various white blood cell types and levels. CSF analysis is mainly used to distinguish meningoencephalitis from other conditions, such as blood toxicity or sepsis. (3) An EEG measures brain electrical signals and is used to assess brain function and diagnose or evaluate meningoencephalitis. (4) A CT scan is an imaging procedure that uses X-rays and a computer to produce detailed images of the body. It can detect problems in the brain or meninges, including clots, swelling, infections, or inflammation. (5) An MRI scan uses a large magnet, radio waves, and a computer to create detailed images, similar to a CT scan. It is preferred for infants and children because it does not involve radiation, making it safer for younger patients.

The primary objectives in the treatment of meningoencephalitis are to address the underlying cause of inflammation and to alleviate associated symptoms. The therapeutic approach may vary based on the specific etiology of the condition. For example, one of the most prevalent forms is herpes meningoencephalitis, which is typically treated with intravenous (IV) administration of the antiviral medication acyclovir for a duration of up to 14 days. It is important to recognize that the effectiveness of antiviral therapy diminishes in the later stages of the disease [6].

In cases of bacterial meningoencephalitis, treatment involves the use of IV antibiotics selected according to the identified causative organism. Additional medications may be prescribed, such as anticonvulsants (e.g., phenytoin or Dilantin) to prevent seizures [7]. Corticosteroids, such as dexamethasone, can help reduce inflammation-related cerebral edema and may also prevent complications such as hearing loss. In some cases, drainage of cerebrospinal fluid (CSF) may be necessary to relieve increased intracranial pressure. Supportive therapy, including pain relievers and sedatives, may also be required to manage discomfort and severe inflammation [1].

## Case presentation

A 20-year-old woman presented to the hospital with a sudden onset of headache, fever, vomiting, and confusion that had persisted for two days. She had no significant past medical history or recent travel. Upon examination, the patient appeared confused and was disoriented to time, place, and person. She also exhibited agitation and aggression towards her family and the hospital staff. Additionally, she showed signs of photophobia and neck stiffness.

Her Glasgow Coma Scale (GCS) score was 14 out of 15 (E4 V4 M6). Both Kernig's and Brudzinski's signs were positive. Upon auscultation, her chest exhibited normal vesicular sounds, with no murmurs detected. Her abdomen was soft and non-tender, and there was no presence of any non-blanching rash.

Blood tests showed significantly elevated levels of inflammatory markers, while cerebrospinal fluid (CSF) glucose levels remained normal, accompanied by elevated protein levels, as detailed in Table 1. The CSF-to-serum glucose ratio was about 0.51. Additionally, both CT and MRI scans of the head were performed, resulting in normal findings. A lumbar puncture revealed xanthochromia and an increased white blood cell count, as described in Table 2. The CSF report, presented in Table 3, identified the causative organism as meningococcal group B.

**Table 1:** Laboratory investigations. Laboratory findings indicate elevated inflammatory markers, including a raised white cell count and C-reactive protein, alongside normal CSF glucose and elevated CSF protein levels. CSF: cerebrospinal fluid, eGFR: estimated glomerular filtration rate, INR: international normalized ratio.

Investigation	Units	Reference range	Result
Hemoglobin	g/L	115-165	120
Mean cell volume	fL	80-99	88
White cell count	x10^9^/L	4.0-11.0	23.9
Neutrophil count	x10^9^/L	1.50-7.50	22.56
Lymphocyte count	x10^9^/L	1.00-4.00	0.58
Platelet count	x10^9^/L	140-400	294
Alanine transaminase	U/L	10-49	9
Total bilirubin	μmol/L	0-21	3
Serum folate	μg/L	10-291	4.7
Vitamin B12	ng/L	211-911	375
Thyroid-stimulating hormone	mlU/L	0.55-4.78	2.4
Creatinine	μmol/L	60-120	49
eGFR	ml/min/1.73 m²	>90	>90
Sodium	mmol/L	133-146	142
Potassium	mmol/L	3.5-5.3	4.1
Urea	mmol/L	2.5-7.8	4.9
Adjusted calcium	mmol/L	2.12-2.51	2.21
Phosphate (inorganic)	mmol/L	0.80-1.50	1.08
Albumin	g/L	35-50	36
Alkaline phosphatase	U/L	30-130	89
Total protein	g/L	57-82	63
C-reactive protein	mg/L	0-10	263
Prothrombin time	seconds	12.0-15.0	13.7
INR	-	-	1.08
Serum glucose	mmol/L	3.5-5.5	8.4
CSF glucose	mmol/L	2.5-4.4	4.3
CSF protein	g/L	0.15-0.45	0.94

**Table 2:** Laboratory findings of cerebrospinal fluid. Cerebrospinal fluid result shows the presence of xanthochromia and raised white cells.

Specimen type	Cerebrospinal fluid
Appearance	Bloodstained fluid
Red cells	1510 x 10^6^/L
White cells	614 x 10^6^/L
Polymorphs	52%
Lymphocytes	48%
Gram stain	Organisms not seen
Net bilirubin absorbance	0.008
Net oxyhemoglobin absorbance	0.006
Cerebrospinal fluid spectrophotometric scan	The interpretation of the xanthochromia assumes
Culture	No growth on primary culture

**Table 3:** Bacteriology reference laboratory report (cerebrospinal fluid). Bacteriology report indicates presence of the meningococcal group B in cerebrospinal fluid.

Specimen type	Cerebrospinal fluid
Meningococcal screening PCR	Detected by PCR
Meningococcal genogroup B DNA	Detected by the siaD gene group-specific PCR

Empirical antimicrobial therapy was initiated with intravenous (IV) ceftriaxone at a dose of 2 g twice daily, along with IV dexamethasone at 9.9 mg twice daily and IV acyclovir at 580 mg three times daily, each administered for five days. After confirming the presence of *Neisseria meningitidis* serogroup B in the cerebrospinal fluid (CSF) culture, the antibiotic regimen was streamlined to IV ceftriaxone at 2 g twice daily. The patient continued this regimen for an additional four days during hospitalization and was subsequently discharged with instructions to continue ceftriaxone at the same dose for another 10 days. This resulted in a total of 14 days of targeted antimicrobial therapy.

The patient responded well to treatment, as evidenced by the improvement in fever and mental state. A series of repeated blood tests over the course of several days showed a decrease in inflammatory markers, with the CRP dropping to 108 and then 49, eventually returning to a normal level. Ultimately, the patient successfully recovered from meningoencephalitis after completing the full course of antibiotics.

## Discussion

Xanthochromia without hemorrhage

Xanthochromia, the yellow discoloration of cerebrospinal fluid (CSF), is primarily used to diagnose subarachnoid hemorrhage (SAH), particularly when neuroimaging is negative or inconclusive. It usually results from hemoglobin breakdown, especially the breakdown of bilirubin. However, xanthochromia is not specific to hemorrhage. Elevated CSF protein (≥1.5 g/L), systemic hyperbilirubinemia, central nervous system infections, and neoplastic infiltration can also cause this finding [8,9]. In our patient, both non-contrast computed tomography (CT) and magnetic resonance angiography (MRA) showed no intracranial hemorrhage or vascular abnormality. Given the absence of radiological evidence of bleeding and a clinical picture consistent with meningoencephalitis, xanthochromia is more likely due to inflammatory or protein-related changes. This underscores the importance of interpreting xanthochromia within the full clinical and laboratory context, rather than attributing it solely to vascular causes.

Normal CSF glucose in bacterial infection

Low cerebrospinal fluid (CSF) glucose is a classic indicator of bacterial meningitis, resulting from bacterial metabolism and impaired glucose transport. However, a normal CSF glucose level does not rule out the diagnosis. Up to one-third of bacterial meningitis cases may initially present with normal glucose, especially early in the illness or after partial antibiotic treatment [10,11]. This pattern is seen in *Neisseria meningitidis* infections, where CSF glucose can remain normal despite severe symptoms [12]. Viral meningoencephalitis typically shows normal glucose, which can complicate differentiation from bacterial causes. CSF glucose should always be interpreted alongside other clinical and laboratory findings. Empirical antimicrobial therapy should not be delayed solely due to a normal glucose result.

Elevated CSF protein

Elevated cerebrospinal fluid (CSF) protein indicates blood-brain barrier disruption and intrathecal inflammation. In bacterial meningoencephalitis, protein levels often exceed 1 g/L, while viral infections usually cause moderate increases [3]. In this case, the patient's CSF protein was above the normal range (0.15-0.45 g/L) but below 1 g/L, making bacterial meningitis less likely. This intermediate elevation may reflect early or partially treated bacterial infection, or a viral process affecting both the meninges and brain. Even modest increases in protein intake can cause xanthochromia, especially in the presence of other inflammatory changes [13]. Although the protein level did not reach the typical threshold for bacterial meningitis, it was still consistent with infectious meningoencephalitis.

## Conclusions

This case presents three critical diagnostic points that must be emphasized. First, xanthochromia in cerebrospinal fluid (CSF) cannot be attributed solely to subarachnoid hemorrhage, especially when CT and MRA scans reveal no signs of bleeding. It is essential to consider inflammatory or protein-related causes as well. Second, a normal CSF glucose level does not eliminate the possibility of infection, as later microbiological testing may clearly demonstrate. Lastly, a moderately elevated CSF protein level, even below the typical threshold for bacterial meningitis, can strongly support the diagnosis when assessed in conjunction with other clinical findings.

Clinically, this case underscores the importance of initiating empirical antimicrobial therapy based on clinical suspicion and doing so promptly. CSF results are then utilized to confirm and refine the diagnosis; in this case, the causative agent was definitively identified as meningococcal group B through PCR testing. In situations where similar CSF findings occur without bacterial identification, it is imperative to explore both infectious and inflammatory causes. Acknowledging these diagnostic nuances equips clinicians to interpret atypical CSF profiles accurately, ensuring timely and effective management of meningoencephalitis.
